# Whole genome sequence and comparative genomic analysis of multidrug-resistant *Staphylococcus capitis* subsp. *urealyticus* strain LNZR-1

**DOI:** 10.1186/s13099-014-0045-x

**Published:** 2014-12-20

**Authors:** Xiaoxia Li, Min Lei, Yanli Song, Kunwei Gong, Ling Li, Hongyan Liang, Xiaofeng Jiang

**Affiliations:** Department of Clinical Biochemistry Laboratory, The Fourth affiliated Hospital of Harbin Medical University, Harbin, China; Department of Clinical Medical Laboratory, The Fifth Hospital of Harbin, Harbin, China

**Keywords:** *Staphylococcus capitis* subsp. *urealyticus*, Multidrug-resistant, Genome sequencing, Comparative genomic analysis

## Abstract

**Background:**

*Staphylococcus capitis* is an emerging opportunistic pathogen of humans, and found as a colonizer of the human gut. Here, we report a case of *S. capitis* subsp. *urealyticus* infection. The strain LNZR-1 was isolated from the blood culture of a patient with sigmoid colon cancer. It was found to be resistant to some important antibiotics, such as linezolid, a highly effective antimicrobial against clinically important *Staphylococci* pathogens. However, data on the genetic resistance mechanisms in *S. capitis* subsp. *urealyticus* are only sparsely available.

**Results:**

The draft genome of *S. capitis* subsp. *urealyticus* strain LNZR-1 was sequenced by using next-generation sequencing technologies. Sequence data assembly revealed a genome size of 2,595,865 bp with a G + C content of 32.67%. Genome annotation revealed the presence of antibiotic resistance genes conferring resistance against some of the tested antibiotics as well as non-tested antibiotics. The genome also possesses a lot of genes that may be related to multidrug resistance. Whole genome comparison of the LNZR-1 with five other *S. capitis* strains showed that some functional regions are highly homologous between the six assemblies made herein. The LNZR-1 genome has high similarity with the genomes of the strains VCU116 and CR01, although some short stretches present in the genomes of strains VCU116 and CR01 were absent in the strain LNZR-1.

**Conclusions:**

The presence of a plethora of genes responsible for antibiotic resistance suggests that strain LNZR-1 could present a potential threat to human health. The comparative genomic analysis of *S. capitis* strains presented in this study is important for better understanding of multidrug resistance in *S. capitis*.

**Electronic supplementary material:**

The online version of this article (doi:10.1186/s13099-014-0045-x) contains supplementary material, which is available to authorized users.

## Background

*Staphylococcus capitis* are Gram positive cocci belonging to the Coagulase-Negative Staphylococci group (CoNS) that is frequently found on the human skin and mucosa [1,2] and even in the human gut [3]. Although infection caused by this species is rare compared with *S. aureus*, infection cases associated with *S. capitis* increase gradually [4]. Recent reports indicate its emergence as a significant pathogen causing nosocomial and bloodstream infections, meningitis, prosthetic valve endocarditis, and late-onset sepsis [4-7]. This bacterium is a subtype of CoNS and the pathogenesis of *S. capitis* is mainly due to its ability to produce a slimy biofilm, enabling it to adhere to the medical devices such as prosthetic valves and catheters; this makes them difficult to be controlled or cleared by immune responses or antibiotic therapy [7].

The *S. capitis* subsp. *urealyticus* strain LNZR-1 described herein was isolated from the blood culture of a patient with sigmoid colon cancer. Antimicrobial susceptibility assay revealed that it was resistant to some important antibiotics, such as linezolid. In order to elucidate the molecular mechanisms behind the multidrug resistance of *S. capitis* subsp. *urealyticus* LNZR-1 clone, here, we report the sequencing and annotation of its genome, together with a functional level genomic comparison with other important *S. capitis* strains, namely QN1 [8], CR01 [9], VCU116, C87 and SK14.

## Methods

### Strain information and growth conditions

The blood samples were collected from a patient with sigmoid colon cancer from the Fourth Affiliated Hospital of Harbin Medical University, in March 2013. *S. capitis* subsp. *urealyticus* strain LNZR-1 was isolated after cultivation. It is a Gram positive, coccus-shaped bacterium growing on 5% sheep blood enriched Columbia agar (BioMérieux, Marcyl’Etoile, France) at 37°C. Cell morphology, motility and sporulation were examined by using scanning electron microscopy.

### Genomic DNA extraction and 16S rRNA gene PCR

Late log-phase cells were harvested and lysed with EDTA and lysozyme, followed by proteinase K and RNase digestion. Genomic DNA was extracted using a DNeasy Blood & Tissue Kit (Qiagen, Germany) according to the manufacturer’s recommended protocol. Agarose gel (0.7%) electrophoresis was used to evaluate the genomic DNA purity and the concentration was measured using a NanoDrop 1000 Spectrophotometer (Thermo Fisher Scientific, USA). The genomic DNA was stored at -20°C. Strain LNZR-1 was identified by 16S rRNA gene sequencing as described earlier [6]. PCR amplification was performed by using primers 27 F (5′-AGAGTTTGATCCTG GCTCAG-3′) and 1500R (5′-AGAAAGGAGGTGATCCAGGC-3′). Agarose gel (1%) electrophoresis was used to separate amplified PCR fragments which were subjected to sequencing of the 16 s rRNA gene. Phylogenetic analysis was conducted based on the 16S rRNA nucleotide sequence. The representative 16S rRNA nucleotide sequence of strain LNZR-1 was compared against the most recent release of the EzTaxon-e database [10]. Phylogenetic inferences were made using Neighbor-joining method based on Tamura-Nei model within the MEGA 6.06 [11].

### Antimicrobial susceptibility testing

Antimicrobial susceptibility was determined by the disk diffusion method on Mueller-Hinton agar recommended by the Clinical and Laboratory Standards Institute guidelines [12]. The following antimicrobial agents were tested: sulfamethoxazole, gentamicin, oxacillin, tetracycline, linezolid, clindamycin, ciprofloxacin, cefoxitin, cefazolin, cefuroxime and vancomycin. The other reference strains used for this study were *Escherichia coli* ATCC 25922 and *Klebsiella pneumoniae* ATCC 700603.

### Genome sequencing, assembly and annotation

The genome of *S. capitis* subsp. *urealyticus* strain LNZR-1 was sequenced using a standard run of Illumina HiSeq 2000 sequencing technology which generated paired-end libraries (500-bp insert size) according to the manufacturer’s instructions. Clean reads were assembled into scaffolds using Velvet version 1.2.07 [13], and Post-Assembly Genome Improvement Toolkit (PAGIT) was used to extend the initial contiguous sequences (contigs) and to correct sequencing errors [14]. Open reading frames (ORFs) were identified using Glimmer version 3.0 [15]. Transfer RNAs and ribosomal RNA genes rRNAs were detected by tRNAscan-SE [16] and RNAmmer 1.2 software [17], respectively. The genome was annotated using the RAST (Rapid Annotation using Subsystem Technology) server [18]. The classification of some predicted genes and pathways was analyzed using the Clusters of Orthologous Groups of proteins (COGs) [19] and Kyoto Encyclopedia of Genes and Genomes (KEGG) [20] databases. Functional annotation was also performed by using public database of National Centre for Biotechnology Information (NCBI).

### Initial comparative genomic analysis

For comparative analysis, we downloaded the reference genome sequences of the closest genetic relatives of strain LNZR-1 and representative strains from the NCBI database: *S. capitis* C87 (ACRH00000000), *S. capitis* SK14 (ACFR00000000), *S. capitis* CR01 (CBUB000000000), *S. capitis* VCU116 (AFTX00000000) and *S. capitis* QN1 (AJTH00000000). Mauve in the progressive mode was used for whole genome comparison [21].

Orthology identification was performed by using NCBI blastp 2.2.25+ with default parameters. Then, bidirectional best hits (BBHs) among proteins from different strains were identified. Furthermore, an identity threshold over a given alignment length to define orthologous genes was applied. Score Ratio Values (SRVs) of BBHs between two genes was calculated with the following formula:

$$ SRVs=\frac{Bits(AB)+ Bits(BA)}{Bits(AA)+ Bits(BB)} $$

Bits (AB) means bits score when using gene A as query while B as database; Bits (BA) means bits score when using gene B as query while A as database; Bits (AA) or Bits (BB) are bits score when using gene A or B to align with itself, respectively. BBHs with SRVs no less than 0.3 was considered as one candidate orthology group (SRVs Table in Additional file 1: Table S1).

## Quality assurance

Biochemical features were tested by using Vitek2 Compact (bioMérieux, Marcy l’Etoile, France). Positive reactions were observed for arginine dihydrolase 1, L-lactate alkalinization, bacitracin resistance, mannose, growth in 6.5% NaCl, O/129 resistance (comp.Vibrio.) and optochin resistance. Negative reactions were obtained for D-amygdalin, phosphatidylinositol phospholipase c, D-xylose, beta-galactose, Ala-Phe-Pro arylamidase, alpha-galactosidase, cyclodextrin, L-aspartate, beta- galactopyranosidase, alpha-mannosidase, phosphatase, leucine arylamidase, L-proline arylamidase, beta-glucuronidase, L-pyrrolidonyl-arylamidase, beta-glucuronidase, alpha-galactosidase, alanine arylamidase, tyrosine arylamidase, D-sorbitol, urease, D-galactose, D-ribose, lactose, N-acetyl-D-glucosamine, methyl-B-D-glucopyranoside, D-maltose, novobiocin resistance, D-mannitol, pullulan, D-raffinose, salicin, D-trehalose and sucrose. Based on the morphological and biochemical characterization, the strain LNZR-1 was identified as *S. capitis*. Bioinformatics assessment of potential contamination of the genomic library by allochthonous microorganisms was achieved using the BLAST non-redundant database.

## Initial findings

### Identification of strain LNZR-1

**Figure 1:**
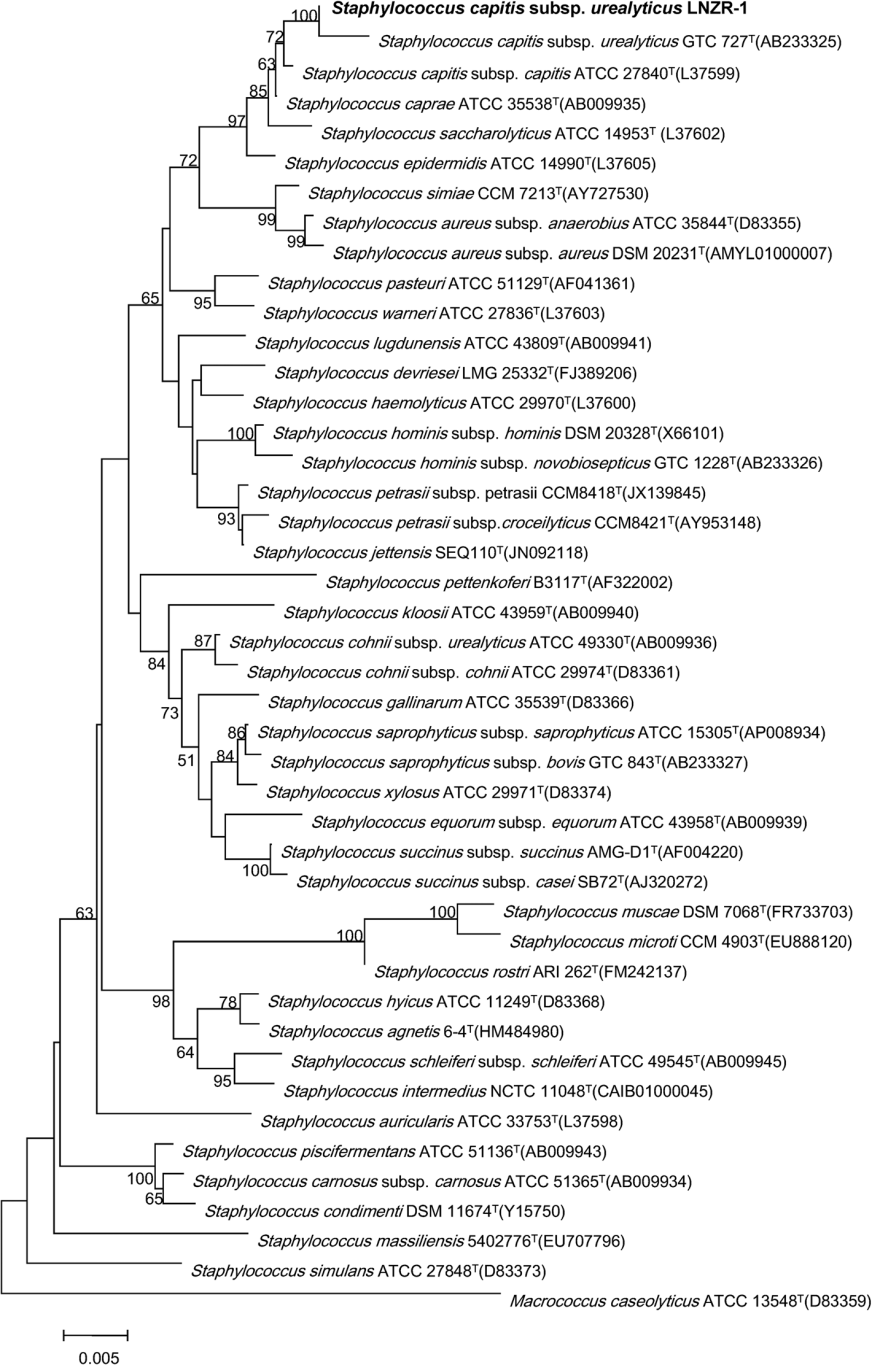
**Phylogenetic tree highlighting the position of** ***S. capitis*** **subsp.** ***urealyticus*** **strain LNZR-1 relative to other type strains within the genus** ***Staphylococcus.*** The strains and their corresponding GenBank accession numbers for 16S rRNA genes are shown following the organism names. Numbers at the branching nodes are percentages of bootstrap values based on 1,000 replications. Bootstrap values greater than 50% are shown at the branch points. *Macrococcus caseolyticus* ATCC 13548^T^ was used as an out group. The scale bar represents 0.005 substitutions per nucleotide position.

Cells of strain LNZR-1 are cocci, 0.7 to 1.2 μm in diameter, occurring predominantly singly or in pairs (Additional file 2: Figure S1). To assess the purity of strain LNZR-1, the 16S rRNA gene sequence of strain LNZR-1 was aligned with sequences of other members of the genus *Staphylococcus* retrieved from the EzTaxon database. Phylogenetic tree indicated the taxonomic status of strain LNZR-1 clearly classified into the same branch with species *S. capitis* subsp. *urealyticus* GTC 727^T^ (Figure 1).

### Genomic features of S. capitis subsp. urealyticus LNZR-1

**Figure 2:**
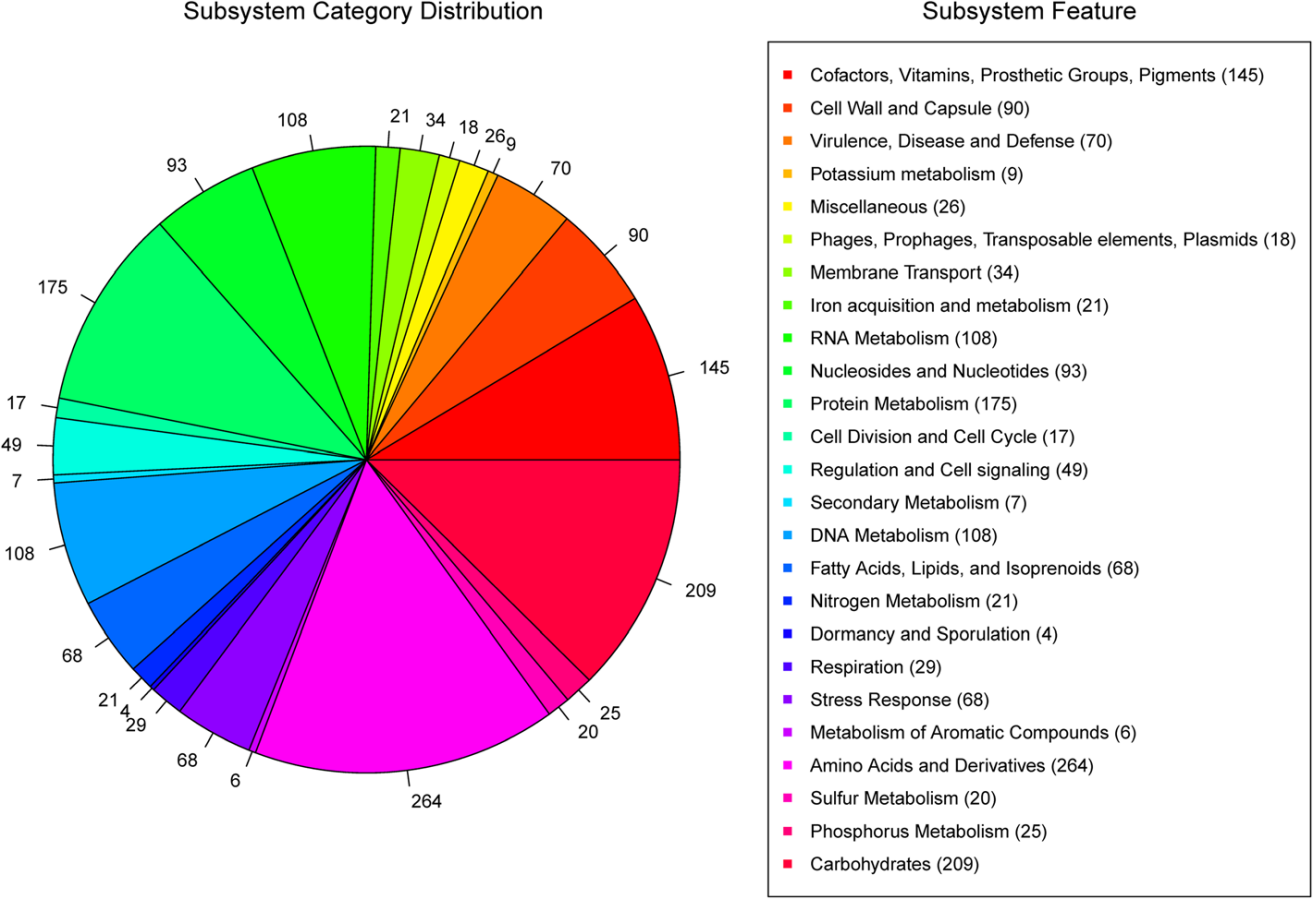
**Subsystems distribution statistic of** ***S. capitis*** **subsp.** ***urealyticus*** **strain LNZR-1 based on genome annotations performed according to RAST server.** The pie chart presents the abundance of each subsystem category and the count of each subsystem feature is listed in parentheses at the chart legend.

**Figure 3:**
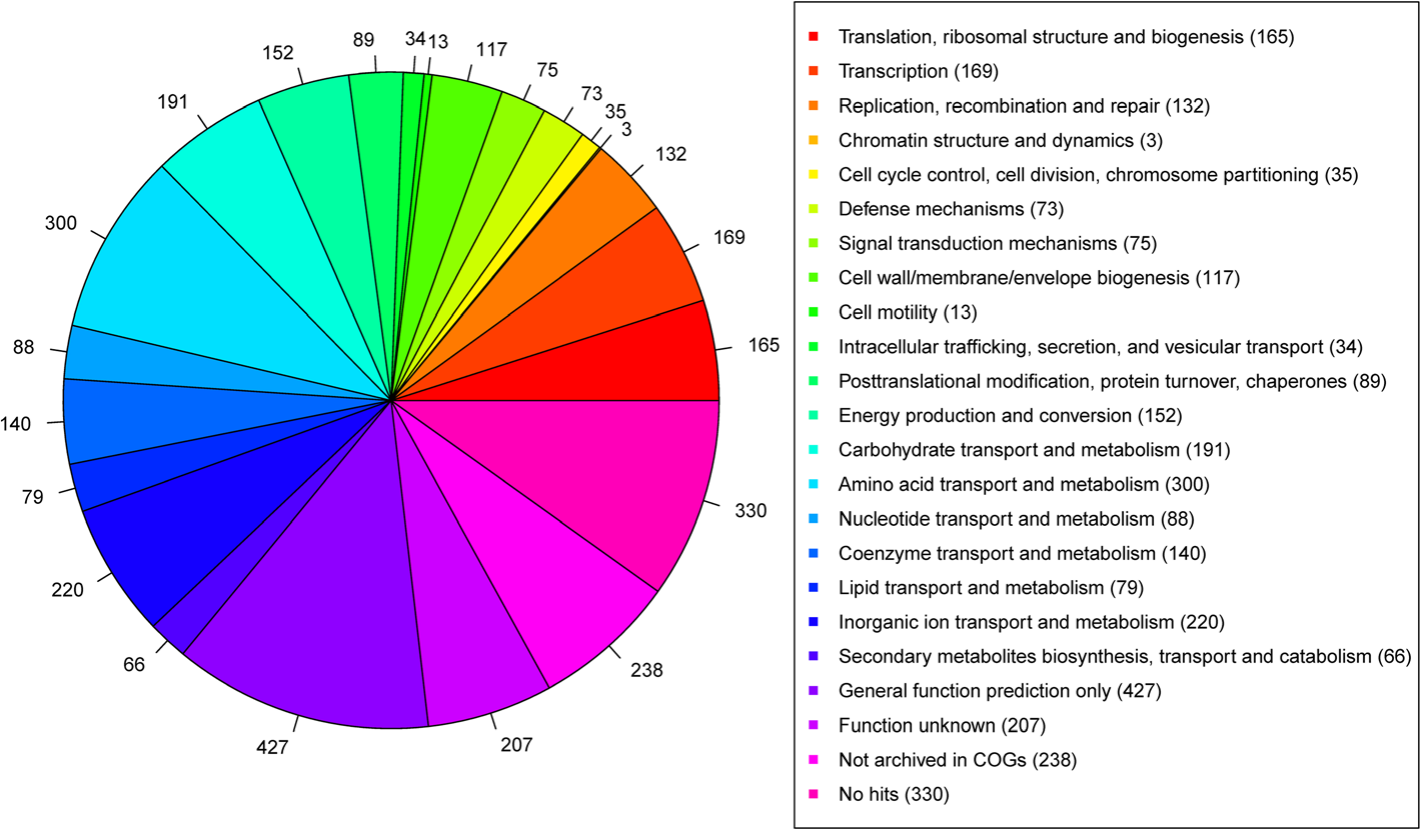
**COGs distribution of** ***S. capitis*** **subsp.** ***urealyticus*** **strain LNZR-1.** Statistics of annotated genes for LNZR-1 were based on COG database.

A total of 2,214,438 available reads were filtered from 5,619,015 raw reads. Quality control was performed with following criteria: reads that contained more than one N bases were removed. Reads that contained more than 50 bases with low quality (Q30) were removed. Reads with more than 10 bases with low quality (Q30) or N bases in the tail of the reads were trimmed. For sequences which lost their mated reads were considered as single reads and were not used in the downstream analysis (Additional file 1: Table S1). 177 initial contigs (best kmer length 57) was assembled by Velvet; then 90 prolonged contigs were assembled based on PAGIT flow (PAGIT is just a flow and it actually does not provide any practically available scripts or programs). The assembled genome of *S. capitis* subsp. *urealyticus* revealed a genome size of 2,595,865 bp and a G + C content of 32.67% (90 scaffolds). The largest contig consisted of 319,806 bp and the length of N50 contig was 66,677 bp. These scaffolds contain 2430 coding sequences (CDSs), 8 tRNAs (excluding 1 Pseudo tRNA) and 2 incomplete rRNA operons (1 small subunit rRNA and 1 large subunit rRNA). The properties and the statistics of the genome are summarized in Table 1. RAST server based annotation of the whole genome describes the subsystem distribution of strain LNZR-1 (Figure 2). Genes responsible for amino acids and derivatives (264 ORFs), carbohydrates (209 ORFs), and protein metabolism (175 ORFs) were abundant among the subsystem categories. 1934 genes were categorized into COGs functional groups (including putative or hypothetical genes, Figure 3). For COGs distribution, R (general function prediction only; 427 ORFs), E (amino acid transport and metabolism; 300 ORFs), P (inorganic ion transport and metabolism; 220 ORFs), S (function unknown; 207 ORFs), and G (carbohydrate metabolism and transport; 191 ORFs) were abundant categories (>10% of total COGs matched counts).

**Table 1:** **Summary of the annotated genome**

**Attribute**	**Genome (total)**
G + C content (bp)	848,103
Coding region (bp)	2,226,123
Total genes	2,461
RNA genes	10
Protein-coding genes	2,430
Genes assigned to COGs	1,934
Genes with signal peptides	124
Genes with transmembrane helices	638

### Antibiotic resistance profile

The *in vitro* antibiotic sensitivity tests demonstrated that this strain is susceptible to vancomycin and sulfamethoxazole, and resistant to tetracycline, gentamicin, ampicillin, methicillin, linezolid, clindamycin, cefoxitin, cefazolin, cefuroxime and ciprofloxacin. To gain insights into the genomic basis for the observed antibiotic resistance traits, the genome was searched for specific genes known to confer antibiotic resistance. The results revealed the presence of antibiotic resistance genes conferring resistance against some of the tested antibiotics as well as non-tested antibiotics (Table 2). MDR-type ABC transporters, multidrug and toxin extrusion (MATE) family efflux pumps and multidrug major facilitator superfamily (MFS) transporters were also detected in the genome. Furthermore, 10 putative MarR family transcriptional regulators were found in the genome, which are recognized as a widely conserved group of multiple antibiotic resistance regulators that respond to diverse antibiotics [22].

**Table 2:** **Summary of putative genes in response to antibiotic resistance in the genome of LNZR-1**

**Start**	**Stop**	**Protein product**	**Length**	**Protein name**
187729	188952	WP_030065164.1	407	Methicillin resistance protein FmtA
819346	819768	WP_002454275.1	140	Fosmidomycin resistance protein
2236269	2238524	WP_030058872.1	751	Daunorubicin resistance protein DrrC
1011427	1012635	WP_023351187.1	402	Bicyclomycin resistance protein TcaB
1024323	1025573	WP_030063338.1	416	Methicillin resistance protein
1074692	1076092	WP_030063319.1	466	Quinolone resistance protein
1378210	1379967	WP_000952923.1	585	Methicillin resistance protein
1848720	1850102	WP_030059174.1	460	Quinolone resistance protein
1979421	1980605	WP_030059066.1	394	Tetracycline resistance protein
985388	987451	WP_023351245.1	687	Drug resistance transporter, EmrB/QacA family
927892	928221	WP_002432921.1	109	Multidrug resistance protein SMR
2584152	2584619	WP_002432814.1	155	Multidrug resistance protein SepA
726004	727740	WP_002435897.1	578	Multidrug ABC transporter ATP-binding protein
845428	846168	WP_002453567.1	246	Multidrug ABC transporter ATP-binding protein
927892	928221	WP_002432921.1	109	Multidrug resistance protein SMR
985388	987451	WP_023351245.1	687	Drug resistance transporter, EmrB/QacA family
987464	988111	WP_002432737.1	215	Multidrug efflux protein
1062539	1063714	WP_023351172.1	391	Putative drug transporter
1523272	1524450	WP_030061422.1	392	Multidrug MFS transporter
1549535	1550377	WP_030061365.1	280	Multidrug ABC transporter ATP-binding protein
1923738	1924907	WP_030059115.1	389	Multidrug MFS transporter
2192933	2194378	WP_030058818.1	481	Multidrug MFS transporter
2195936	2197183	WP_030058823.1	415	Multidrug MFS transporter
2515785	2516984	WP_002432900.1	399	Multidrug MFS transporter
2520095	2523274	WP_030056889.1	1059	Multidrug transporter
2582688	2584034	WP_023351326.1	448	Multidrug transporter
2584152	2584619	WP_002432814.1	155	Multidrug resistance protein SepA
2584703	2586145	WP_002453755.1	480	Multidrug MFS transporter
1182425	1183750	WP_023350278.1	441	MATE efflux family protein
177910	178329	WP_002435477.1	139	MarR family transcriptional regulator
991886	992341	WP_002453883.1	151	MarR family transcriptional regulator
1009611	1009976	WP_030063362.1	122	MarR family transcriptional regulator
1399165	1399611	WP_002432941.1	148	MarR family transcriptional regulator
1894346	1894801	WP_002436214.1	151	MarR family transcripitonal regulator
1932506	1932946	WP_002433445.1	146	MarR family transcriptional regulator
1948086	1948445	WP_002433103.1	119	MarR family transcriptional regulator
2424049	2424513	WP_030058661.1	154	MarR family transcriptional regulator
2515376	2515726	WP_002432752.1	116	MarR family transcriptional regulator
2516984	2517424	WP_030056887.1	146	MarR family transcriptional regulator

### Comparative analysis with other S. capitis strains

**Figure 4:**
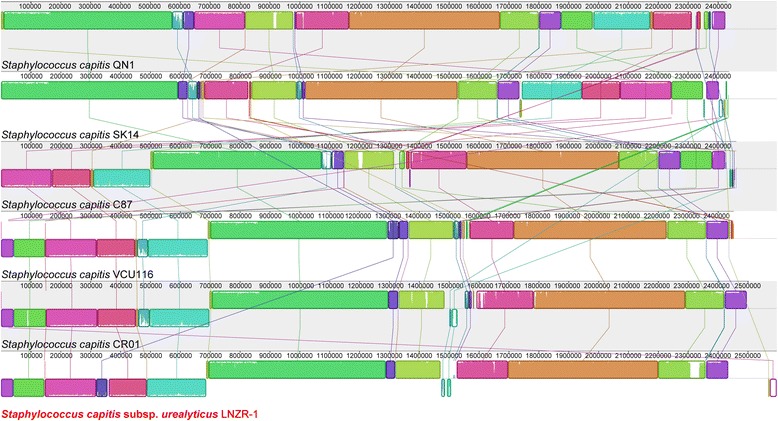
**MAUVE alignment of the genomes of** ***S. capitis*** **subsp.** ***urealyticus*** **LNZR-1,** ***S. capitis*** **C87,** ***S. capitis*** **SK14,** ***S. capitis*** **CR01,** ***S. capitis*** **VCU116 and** ***S. capitis*** **QN1.** MAUVE identifies and aligns regions of local collinearity called locally collinear blocks (LCBs), a region without rearrangement of homologous backbone sequence. LCBs below a genome’s center line are in the reverse complement orientation relative to the reference genome. Lines between genomes trace each orthologous LCB through every genome.

**Figure 5:**
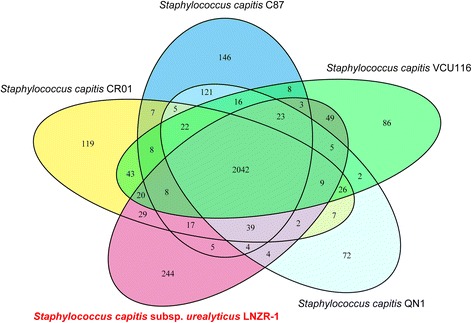
**Venn diagram representing the pan-genome of** ***S. capitis*** **subsp.** ***urealyticus*** **LNZR-1,** ***S. capitis*** **C87,** ***S. capitis*** **CR01,** ***S. capitis*** **VCU116 and** ***S. capitis*** **QN1.** Numbers inside the Venn diagram indicate the number of genes found to be shared among the indicated genomes.

Whole genome comparison of the LNZR-1 with *S. capitis* C87, *S. capitis* SK14, *S. capitis* CR01, *S. capitis* VCU116 and *S. capitis* QN1 showed that some functional regions are highly homologous between the six assemblies (Figure 4). The LNZR-1 genome has high similarity with VCU116 and CR01, although some short stretches present in the genome of VCU116 and CR01 were absent in LNZR-1. Furthermore, LNZR-1, C87, CR01, VCU116 and QN1 revealed a large number of orthologous genes (Figure 5). Venn diagram indicates the presence of a large core-genome. These five *S. capitis* strains shared 2042 CDS in the genome. A particular overlap between C87 and QN1 became evident; these two chromosomes shared 121 orthologous CDS exclusively. The chromosome of LNZR-1 overlapped less with the C87 and QN1, which shared 5 and 4 exclusively orthologous CDS, respectively. In addition, 244 CDS from the LNZR-1 genome were classified as unique.

## Future directions

In recent times, the alarming spread of antibiotic resistance has severely limited the treatment options for nosocomial infections. Currently, the antibiotics linezolid, vancomycin and daptomycin form an empirical therapy towards the control of serious infections caused by *Staphylococci*. However, the overuse of these antibiotics could have resulted in the emergence of multidrug resistant bacteria. Despite untiring efforts directed at the analyses of intermediately resistant clinical isolates, the explicit mode of the development of resistance to these antimicrobials remains obscure. Further studies by using high-throughput mRNA sequencing (RNA-Seq) experiments to explore differential RNA expression levels under selective antibiotic pressures are warranted. Further studies are required to elucidate the resistance mechanisms, and information on these mechanisms could potentially aid in antibiotic development.

### Ethics approval

This research was approved by the Research Ethics Committee of the Fourth affiliated Hospital of Harbin Medical University, and informed consent was obtained from the patient.

## Availability of supporting data

This Whole Genome Shotgun project has been deposited at DDBJ/EMBL/GenBank under the accession JGYJ00000000. The version described in this paper is version JGYJ01000000.

## Additional files

Additional file 1: Table S1. Scanning electron micrograph of cells of *Staphylococcus capitis* subsp. *urealyticus* strain LNZR-1. Additional file 2: Figure S1. The summary of SRVs.
