# Supplementary figures and images for: Whole genome sequence and comparative genomic analysis of multidrug-resistant Staphylococcus capitis subsp. urealyticus strain LNZR-1
Source: Gut Pathog. 2014 Dec 20;6:45. doi: 10.1186/s13099-014-0045-x (PMC4310196; doi:10.1186/s13099-014-0045-x)

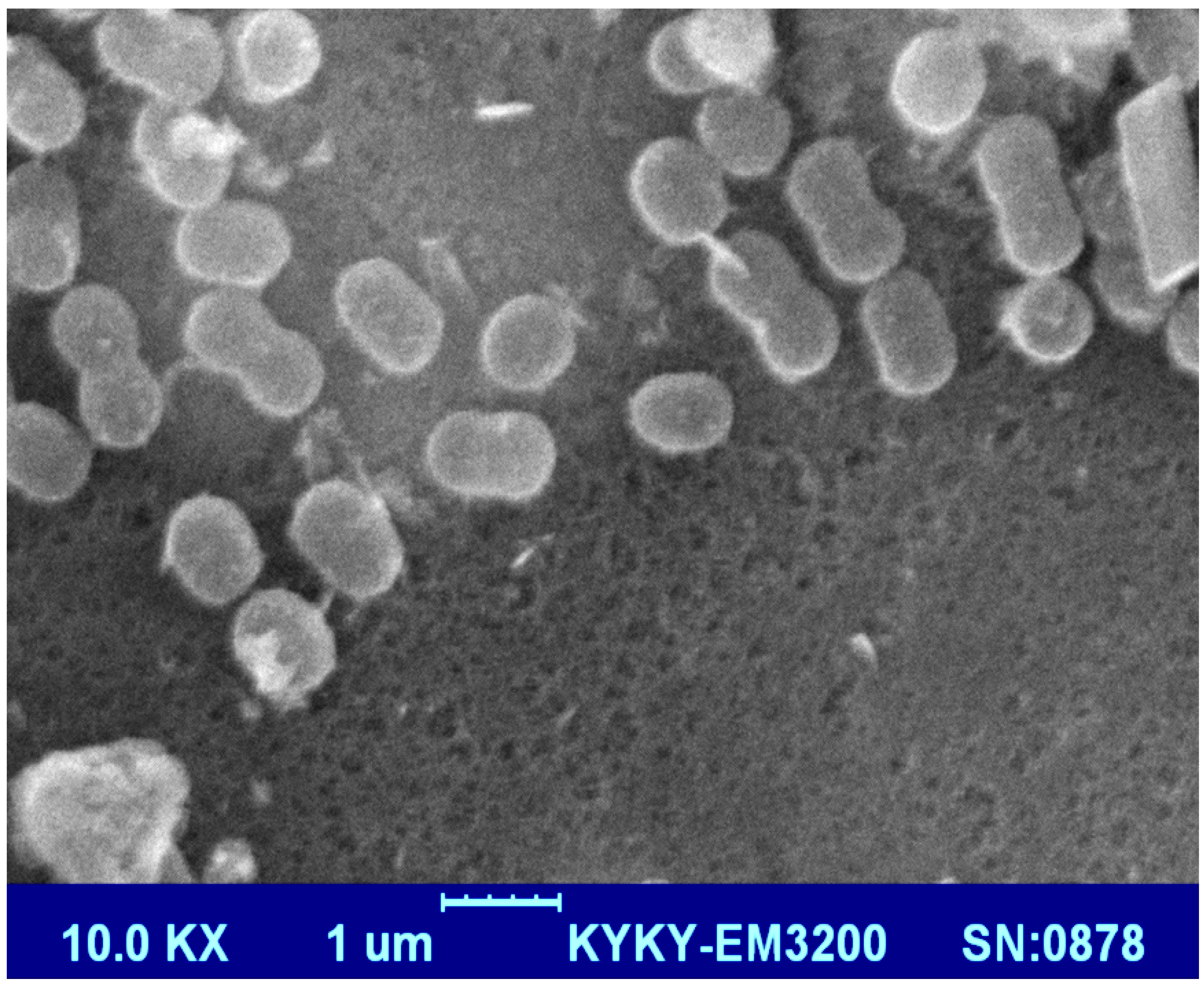

Supplement: Additional file 2: Figure S1. — The summary of SRVs. [file 13099_2014_45_MOESM2_ESM.tiff]
